# Bio-Based pH Indicator Films for Intelligent Food Packaging Applications

**DOI:** 10.3390/polym14173622

**Published:** 2022-09-01

**Authors:** Iulia Păușescu, Diana-Maria Dreavă, Ioan Bîtcan, Raluca Argetoianu, Diana Dăescu, Mihai Medeleanu

**Affiliations:** Faculty of Industrial Chemistry and Environmental Engineering, University Politehnica Timişoara, Carol Telbisz 6, 300001 Timisoara, Romania

**Keywords:** bio-inspired flavylium dye, biopolymer, biodegradable, pH-sensitive film, chitosan, cellulose, starch, PVA

## Abstract

The widespread concerns about the environmental problems caused by conventional plastic food packaging and food waste led to a growing effort to develop active and intelligent systems produced from renewable biodegradable polymers for food packaging applications. Among intelligent systems, the most widely used are pH indicators, which are generally based on a pH-sensitive dye incorporated into a solid support. The objective of this study was to develop new intelligent systems based on renewable biodegradable polymers and a new bio-inspired pH-sensitive dye. The structure of the dye was elucidated through FT-IR and 1D and 2D NMR spectroscopic analyses. UV-VIS measurements of the dye solutions at various pH values proved their halochromic properties. Their toxicity was evaluated through theoretical calculations, and no toxicity risks were found. The new anthocyanidin was used for the development of biodegradable intelligent systems based on chitosan blends. The obtained polymeric films were characterized through UV-VIS and FT-IR spectroscopy. Their thermal properties were assessed through a thermogravimetric analysis, which showed a better stability of chitosan–PVA–dye and chitosan–starch–dye films compared to those of chitosan–cellulose–dye films and the dye itself. The films’ sensitivity to pH variations was evaluated through immersion in buffer solutions with pH values ranging from 2 to 12, and visible color changes were observed.

## 1. Introduction

The food industry is confronted with two major problems that have raised concerns at the global level: the accumulation of synthetic plastic and food waste. Petroleum-based materials’ availability is limited, and their waste disposal is problematic due to poor degradability, leading to the accumulation of plastic materials and plastic pollution [1,2]. Food waste, which accounts for 20 to 40% of the global production depending on the type of food, is gaining attention not only because of its ethical and economic aspects, but also because it depletes the environment of limited natural resources, which is even more worrying in view of the projected increase in the worldwide population [3,4]. Advances in food packaging materials seem to be the solution to both problems. Other significant aspects that strongly influence food packaging development are food safety regulations, which require constant upgrades and updates, as well as consumers’ demands for safe, functional, and convenient packaging to respond to their modern lifestyles [5,6,7]. The above-mentioned reasons led to an increased research interest from both the scientific and industrial communities to develop more advanced types of packaging: active and intelligent systems produced from renewable biodegradable polymers [8,9].

Intelligent food packaging systems are designed to monitor and inform on the condition of food and/or the environment surrounding the food during transport and storage. These systems have the ability to track and record critical parameters for food quality, thus providing dynamic feedback to the producer, retailers, and consumers [10]. Sensors, indicators, and identification systems are the main components of intelligent systems. An indicator incorporated into the matrix of the packaging materials responds to different stimuli, usually through a visual change [6,11]. Chromogenic chemosensors, namely, colorimetric pH indicators, which are generally based on pH-sensitive dyes incorporated into a solid support, are mostly used in food packaging materials because food spoilage is frequently accompanied by pH changes [12,13].

Among natural dyes, which have the benefit of being safe and ecofriendly, anthocyanins are the most researched class. Their ability to change color upon pH modification enables them to be used as sensing dyes for intelligent food packaging materials [14,15,16]. Anthocyanins exhibit halochromic properties as a result of the pH-dependent equilibrium reactions of flavylium cations. Despite their enormous potential benefits, anthocyanins are highly reactive molecules that are sensitive to degradation by temperature, light, oxygen, and pH, which affects their stability and coloring properties [17,18]. Moreover, their extraction, separation and purification imply high costs and time-consuming and laborious procedures [19]. To overcome the disadvantages of natural anthocyanins, bio-inspired dyes have been developed to enhance desirable and specific properties through sustainable, low-cost synthetic routes [20].

The most studied biopolymers for food packaging applications are polysaccharides (starch and cellulose derivatives, chitosan, and alginates), lipids (bees and carnauba wax, free fatty acids), proteins (casein, whey, and gluten), poly hydroxybutyrates (PHB), polylactic acid (PLA), poly caprolactone (PCL), polyvinyl alcohol (PVA), poly butylene succinate, and their biopolymer blends [11,21,22]. Unfortunately, biopolymers present some important drawbacks: poor mechanical, thermal, and barrier properties, high hydrophilic properties, and poor processability; even though the addition of plasticizers and compatibilizers has proven to be beneficial, their properties are still not satisfactory for industrial applications [2,21].

To overcome the limitations to their use for food packaging films, some of the most established methods are physical, chemical, and biochemical modifications. To increase the hydrophobicity of biopolymers, the addition of lipids, waxes, and oils is often used, with good results in reducing the water vapor permeability, but, unfortunately, with an increase in opacity, uneven surfaces, and poorer mechanical properties [22,23]. One of the most commonly used methods for the development of polymeric films for food packaging is blending, which is a low-cost and easy technique for obtaining films with tailored properties by combining polymers with desired properties. Another method used to improve the properties of biodegradable films is crosslinking, which implies the formation of intra- or intermolecular chemical bonds between the chains of polymers. Through crosslinking, the three-dimensional polymeric networks become more compact and solid [22,24]. By dispersing inert filler materials into the polymeric matrix, a process known as reinforcement, polymer composites or polymers nanocomposites with improved mechanical and barrier properties can be obtained [25,26,27].

Chitosan is the second most abundant biopolymer. It is a biodegradable, biocompatible, non-toxic, and renewable natural polymer, with significant biological properties (antimicrobial, antioxidant, anticancer, anti-inflammatory, hemo-compatible, and hemostatic) and good film-forming properties [28,29]. Chitosan is often blended with other polymers—usually thermoplastic ones—to obtain films with improved properties, which has caused them to be intensively researched for their use in active and intelligent films [30,31].

Polyvinyl alcohol (PVA) is a petroleum-based, biodegradable, water-soluble, semicrystalline, and non-toxic polymer with good biocompatible properties, and it has the ability to form films with a high transparency, remarkable chemical resistance, good mechanical behavior, and adhesive and antistatic properties [29,32].

Starch is one of the most abundant naturally available renewable polysaccharides; it consists of amylose and amylopectin units, where amylose mainly contributes to the film-forming capacity. Starch-based films are water soluble, non-toxic, colorless, biologically absorbable, flexible, and oxygen impermeable; however, their mechanical properties are poor because starch is not thermoplastic [33,34], so the addition of plasticizers or blending with other polymers is necessary to make them suitable for packaging applications [35].

Cellulose is the most abundant biopolymer on Earth and consists of glucopyranosyl units linked with β-1,4. This polysaccharide can be extracted from wood, cotton, and other plants. Its good biodegradability, biocompatibility, and low toxicity make it a promising material for numerous applications, especially as a food packaging material because of its beneficial mechanical properties, significant strength, and low cost [36,37].

The aim of this study was to synthesize a bio-inspired pH-sensitive dye and to incorporate it into renewable biodegradable polymeric matrices to develop pH indicators for food packaging applications. Due to its above-mentioned extraordinary properties and its extensive use as a material for food packaging applications, chitosan was chosen as the main biopolymer for blending, along with PVA, starch, and cellulose, which were selected for the complementarity of their properties to those of chitosan.

## 2. Materials and Methods

### 2.1. Materials

The 4′-hydroxy-3′,5′-dimethoxyacetophenone (97%), 2-hydroxy-4-diethylamino-benz- aldehyde (98%), citric acid (>99.5%), boric acid (H_3_BO_3_, >99.5%), trisodium phosphate (Na_3_PO_4_, 96%), ammonia aqueous solution (25%), polyvinyl alcohol (PVA, Mw 89,000–98,000; 98% + hydrolyzed), chitosan (high molecular weight), starch from potatoes (soluble), and glycerol (99.5%) were purchased from Sigma Aldrich (Steinheim am Albuch, Germany). Sulfuric acid (H_2_SO_4_, 95–97%), acetic acid (CH_3_COOH, 98%), and microcrystalline cellulose were acquired from Merck KGaA (Darmstadt, Germany). Methanol (MeOH, >99%) and diethyl ether (>99%) were purchased from CHIMREACTIV SRL (Bucuresti, Romania).

All chemicals, reagents, and solvents were used as purchased, without further purification, for the synthesis and preparation of samples.

### 2.2. Methods

#### 2.2.1. Synthesis of 7-diethylamino-4′-hydroxy-3′,5′-dimethoxyflavylium Hydrogensulfate

The flavylium dye 7-diethylamino-4′-hydroxy-3′,5′-dimethoxyflavylium was obtained in acidic conditions through a previously described condensation procedure [38]. First, 4′-hydroxy 3′,5′-dimethoxyacetophenone (0.3924 g, 0.002 mol) and 2-hydroxi-4-diethylamino-benzaldehyde (0.3865 g, 0.002 mol) were placed into a round-bottom flask and dissolved in 12 mL of acetic acid 99%; then, 3 mL of sulfuric acid 94% was added. The mixture was magnetically stirred at room temperature for 24 h. The following day, diethyl ether was added, and the formation of a dark-colored precipitate was observed. It was filtered off, further washed with diethyl ether, and dried, resulting in 0.663 g of violet precipitate (yield, η = 73.5%; melting point, m.p. = 195–198 °C).

FT-IR (ATR) cm^−1^: 3070; 2980; 2887; 2480; 1749; 1614; 1556; 1504; 1458; 1367; 1230; 1146; 1097; 1038; 970; 845; 743; 571; 436.

^1^H-NMR (500 MHz, DMSO-d_6_, δ ppm): 1.26 (t, 6H, *J* = 7.0 Hz); 3.72 (q, 4H, *J* = 7.0 Hz); 3.96 (s, 6H); 7.34 (s, 1H); 7.44 (dd, 1H, *J* = 9.4; 2.3 Hz); 7.63 (s, 2H); 7.98 (d, 1H, *J* = 9.4 Hz); 8.18 (d, 1H, *J* = 8.3 Hz); 8.75 (d, 1H, *J* = 8.3).

^13^C-NMR (125 MHz, DMSO-d_6_, δ ppm): 12.9; 45.8; 57.1; 96.3; 106.8; 109.4; 118.2; 119.6; 132.6; 143.8; 149.0; 156.1; 159.2; 166.9.

#### 2.2.2. Film Preparation

- Chitosan–cellulose films

A 2% (*w*/*v*) chitosan solution was prepared by dissolving the appropriate amount of chitosan in 2% (*v*/*v*) aqueous acetic acid. To 100 mL of the obtained solution, 0.2 g and 10 mg of the flavylium dye were added. The solution was magnetically stirred at room temperature until all components were dissolved. The mixture was carefully poured into Petri dishes with a 15 cm diameter, which were placed for 5 min in an exicator saturated in NH_3_ vapors [39]. Afterwards, the Petri dishes were dried in an oven for 48 h at 40 °C. Following the same steps, a film without the dye was prepared. The polymeric films were carefully peeled off and kept in closed containers at room temperature in dark conditions until characterization.

- Chitosan–starch films

The second blend was obtained using a 2% (*w*/*v*) chitosan solution prepared as above and a 5% (*w*/*v*) starch aqueous solution by dissolving the appropriate amount of starch in distilled water. The ratio between the two solutions was 1:1 (*v*/*v*). To 100 mL of this mixture, 0.4 g of glycerol and 10 mg of the flavylium dye were added [40]. The solution was magnetically stirred until the solid was dissolved; then, it was placed into an ultrasonic bath for CO_2_ removal. The films were obtained by pouring the solution into Petri dishes with a 15 cm diameter, which were dried in an oven for 48 h at 40 °C. A film without the dye was prepared according to the same procedure. The polymeric films were carefully peeled off and kept in closed containers at room temperature in dark conditions until characterization.

- Chitosan–PVA films

The third blend was prepared by mixing a 2% (*w*/*v*) chitosan solution and a 10% (*w*/*v*) PVA aqueous solution at a ratio of 3:7 (*v*/*v*) [41]. To 100 mL of the obtained solution, 10 mg of the pH-sensitive dye was added. The solution was magnetically stirred until the solid was dissolved; then, it was placed into an ultrasonic bath for CO_2_ removal. The films were obtained by pouring the solution into Petri dishes with a 15 cm diameter, which were dried in an oven for 48 h at 40 °C. A film without the dye was prepared by following the same procedure. The polymeric films were carefully peeled off and kept in closed containers at room temperature in dark conditions until characterization.

#### 2.2.3. Study of the Halochromic Properties 

The investigation of the halochromic properties of the flavylium dye involved measurements of the UV-VIS spectra of the flavylium dye solutions (7 × 10^−5^ M in methanol:water 1:14) over time at pH values ranging from 2 to 12. The buffer solutions were prepared by following a previously described procedure [42], and the pH values were measured with a Mettler Toledo Seven Compact S210-K (Mettler Toledo, Columbus, OH, USA) at 25 °C.

The colorimetric response to pH changes was assessed by recording the UV-VIS spectra of 1 × 1 cm polymeric films after they had been immersed for 1 h in buffer solutions ranging from 2 to 12, then washed with distilled water and dried at 40 °C for 4 h.

#### 2.2.4. Infrared Spectroscopy (ATR FT-IR)

FT-IR spectra were registered on a Bruker Vertex 70 (Bruker Daltonik GmbH, Bremen, Germany) spectrometer equipped with a Platinium ATR, Bruker Diamond Type A225/Q, in attenuated total reflectance (ATR) mode. The spectra of the flavylium dye and the polymeric films were obtained through the co-addition of 64 scans in the 4000–400 cm^−1^ spectral domain with a resolution of 4 cm^−1^.

#### 2.2.5. UV-VIS Spectroscopy

The UV-VIS absorption spectra were recorded on an Agilent Cary 60 spectrophotometer (Agilent Technologies, Waldbronn, Germany) at 25 °C.

#### 2.2.6. pH Measurements

The pH of the solutions was measured with a Mettler Toledo Seven Compact S210-K (Mettler Toledo, Columbus, OH, USA) at 25 °C.

#### 2.2.7. NMR Analysis

For the NMR analysis, the samples were dissolved in DMSO-d_6_. The NMR spectra (1D NMR: ^1^H, ^13^C, DEPT 135 and 2D NMR: COSY, HQSC, HMBC) were recorded on a Bruker AVANCE III spectrometer (Bruker Daltonik GmbH, Bremen, Germany) operating at 500.0 MHz (^1^H) and 125.0 MHz (^13^C) at 298 K. The chemical shifts δ are reported in ppm versus tetramethylsilane (TMS), and the coupling constants are reported in Hz. For the splitting patterns, the following abbreviations are used: s (singlet), d (doublet), dd (doublet of doublets), t (triplet), and q (quartet).

#### 2.2.8. Thermogravimetric Analysis

The thermograms of the films and dye were recorded by using a TG 209 F1 Libra thermogravimetric analyzer (NETZSCH-Gerätebau GmbH, Selb, Germany). The analyses were carried out in a nitrogen atmosphere from 20 to 600 °C, with a heating rate of 10 °K/min. All data were processed with the Netzsch Proteus Thermal Analysis software version 6.1.0. (NETZSCH-Gerätebau GmbH, Selb, Germany).

The melting point of the dye was determined on a Carl Zeiss melting point apparatus (Carl Zeiss, Oberkochen, Germany) and is uncorrected.

## 3. Results and Discussion

The development of bio-inspired synthetic pH-sensitive dyes could be the solution to the poor chemical and color stability of natural dyes with applications in intelligent packaging materials for the food industry. Synthetic anthocyanidins can be easily obtained through a sustainable chemical route involving acid-catalyzed condensation between acetophenones and substituted salicylaldehydes.

### 3.1. Synthesis and Characterization of the pH-Sensitive Dye

**Figure 1:**
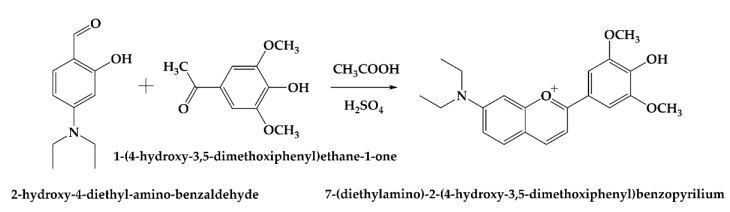
Reaction scheme for the synthesis of the flavylium dye.

In this study, we report the synthesis and characterization of a new bio-inspired flavylium dye with possible applications as a pH-sensitive dye for intelligent packaging systems. The reaction scheme is presented in Figure 1.

The synthetic anthocyanidin was characterized with UV-VIS, FT-IR, and NMR spectroscopy methods. Its thermal stability was evaluated through thermogravimetric analysis.

The FT-IR analysis revealed the presence of the main functional groups and structural elements in the synthesized dye, which were evidenced by the following characteristic absorption bands: 3070 cm^−1^ ($\nu_{CarH}$), 2980 cm^−1^ ($\nu_{CH_{3}}^{as}$), 2887 cm^−1^ ($\nu_{CH_{3}}^{s}$), 1749 cm^−1^ ($\nu_{C=O^{+}}$), 1614, 1556 cm^−1^ ($\nu_{sk,ar“1600”}$), 1504, 1458 cm^−1^ ($\nu_{sk,ar“1500”}$), 1367 cm^−1^ ($\nu_{Car-N}$), 845 cm^−1^ (1,2,3,5-tetrasubstituted benzene ring), 1146 cm^−1^ ($\nu_{COC}^{s}$), 1097, 1038 cm^−1^ ($\nu_{COC}^{as}$). The FT-IR spectra are presented in Supplementary Materials (Figure S1).

NMR analysis confirmed the exact structure of the dye. The 1D NMR (^1^H, ^13^C, DEPT 135) and 2D NMR (HMBC) spectra are presented in the Supplementary Materials, Figures S2–S5. The ^1^H-NMR spectrum revealed signals at 1.26 (CH_3_ from the ethyl groups), 3.72 (N-(CH_2_-CH_3_)_2_), and 3.96 ppm (O-CH_3_), which were specific to aliphatic protons. The signals corresponding to the aromatic protons were found between 7.34 and 8.75 ppm. In the ^13^C-NMR spectrum, aliphatic signals were identified at 12.9 (CH_3_ from the ethyl groups), 45.8 (N-(CH_2_-CH_3_)_2_), and 57.1 ppm (O-CH_3_), while the aromatic carbons were attributed to signals between 96.3 and 166.9 ppm. The most deshielded carbons were those linked to O and N atoms (166.9 ppm C = O^+^, 159.2 ppm (C-O^+^), 156.1 ppm C-N-(CH_2_-CH_3_)_2_, 149.0 ppm C-OCH_3_, 143.8 ppm (C-OH). 

The full NMR analysis is presented in the Supplementary Materials.

The UV-VIS spectra of the dye showed an absorption maximum at 539 nm (Supplementary Materials, Figure S6).

The thermogram of the dye revealed a single degradation step with an inflexion point at 193.8 °C (Supplementary Materials, Figure S7).

### 3.2. Halochromic Properties of the Dye

**Figure 2:**
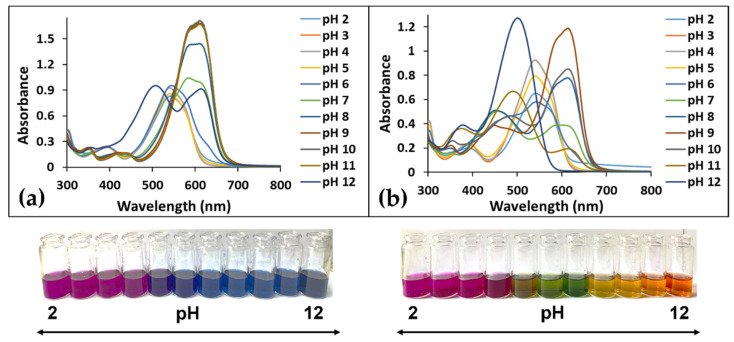
UV-VIS spectra and color of dye species at pH 2–12 after (**a**) 20 min and (**b**) 48 h.

The pH-dependent photochromic properties of the synthesized dye were evaluated through an UV-VIS spectroscopy study. The UV-VIS spectra of the dye solutions at pH values ranging from 2 to 12 were registered over time. The overlaid spectra presented in Figure 2a show the existence of multiple species upon pH changes, as evidenced by the shifting of the absorption maximum and by the color modification in time (Figure 2b).

**Figure 3:**
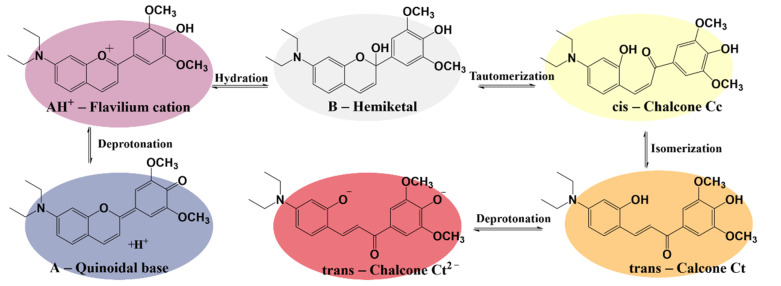
The network of reversible chemical reactions of the bio-inspired dye species upon pH changes.

The synthetic anthocyanidins followed the same well-known network of reversible chemical reactions upon pH changes as the natural ones. Figure 3 presents the main species of the synthesized dye involved in the reversible transformations that occurred under different acidic and basic conditions.

The dye was obtained in its flavylium cation form AH^+^ (λ = 539 nm). This species was stable at pH values below 5. Once the pH rise, there were two competing transformations—a very fast deprotonation of the AH^+^, which led to the quinoidal base A (λ = 586 nm), and a much slower hydration process, which resulted in the colorless hemiketal B. In time, the hemiketal was transformed through tautomerization into a *cis*-chalcone Cc, which, after an isomerization process, gave a *trans*-chalcone Ct. The chalcone species were yellow to orange in color (λ = 418 nm, λ = 456 nm). In very basic conditions, the trans-chalcone was fully deprotonated, resulting in the red-colored Ct^2^^–^ species (λ = 500 nm).

All registered UV-VIS spectra are presented in the Supplementary Materials, Figure S8.

### 3.3. Theoretical Toxicity Evaluation

The toxicity of organic compounds used in food packaging is essential in food security and health. To evaluate the toxicity risk of these compounds, the OSIRIS Property Explorer was used [43]. This is a system that evaluates the risk of mutagenic capacity, tumor generation, and irritating and reproductive effects of tested organic compounds by comparing them with a large set of known structures that emphasize these unwanted properties. Other interesting molecular descriptors calculated are molecular weight, octanol/water partition coefficient (cLogP), solubility (logS), topological polar surface area (TPSA), drug-likeness, and, finally, the drug score, which is a cumulative computed property based on all others.

The above descriptors for malvidin structure (natural compound) and the synthesized dye were calculated using the OSIRIS Property Explorer. The results are presented in Table 1.

Based on these results, there is no toxicity risk for either the natural compound or the synthesized one. On the other hand, none of these compounds can be classified as drug-similar (the drug-likeness was negative, and the overall drug score was very small).

### 3.4. Film Characterization

The bio-based films were characterized with spectroscopic methods (FT-IR and UV-VIS) and thermogravimetric methods.

#### 3.4.1. FT-IR

FT-IR spectroscopy was employed to characterize the presence of the main functional groups of chitosan, cellulose, starch, and PVA in the prepared bio-based polymeric films and to evidence the inclusion of the dye in their matrices.

**Figure 4:**
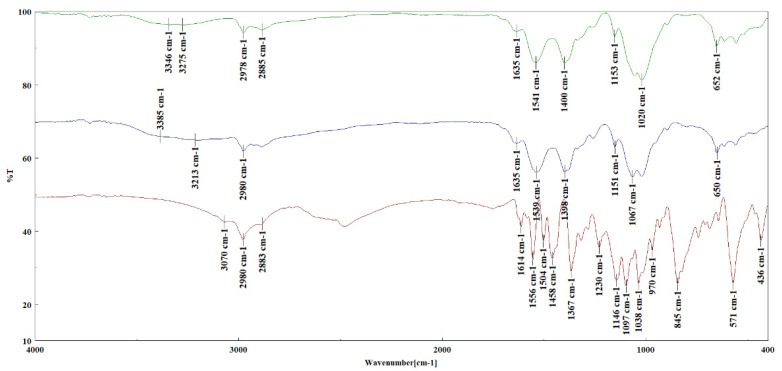
FT-IR spectra of chitosan–cellulose–dye (green), chitosan–cellulose (blue), and dye (red).

Chitosan and cellulose have similar chemical groups (C-H, O-H, C-O); therefore, their vibrational bands are similar and overlapping. The FT-IR spectra of chitosan-cellulose, chitosan–cellulose–dye, and dye are shown in Figure 4. The control chitosan–cellulose film showed absorption bands characteristic of both chitosan and cellulose: 3385, 3213 cm^−1^ ($\nu_{OH}$), 2980 cm^−1^ ($\nu_{CH}^{as}$), 2885 cm^−1^ ($\nu_{CH}^{s}$), 1653 cm^−1^ ($\nu_{C=O}$ - amide I-chitosan), 1539 cm^−1^ ($\delta_{NH_{2}}$ and $\nu_{C-N}$ overlapping), 1151 cm^−1^ ($\nu_{COC}^{s}$), 1067, 1020 cm^−1^ ($\nu_{COC}^{as}$), 650 cm^−1^ ($\gamma_{OH}$). Many of the vibrational bands of the chitosan–cellulose–dye were shifted because of different intra- and intermolecular interactions: 3346, 3275 cm^−1^ ($\nu_{OH}$), 2978 cm^−1^ ($\nu_{CH}^{as}$), 2885 cm^−1^ ($\nu_{CH}^{s}$), 1653 cm^−1^ ($\nu_{C=O}$ - amide I-chitosan), 1541 cm^−1^ ($\delta_{NH_{2}}$ and $\nu_{C-N}$ overlapping), 1153 cm^−1^ ($\nu_{COC}^{s}$), 1067, 1020 cm^−1^ ($\nu_{COC}^{as}$), 652 cm^−1^ ($\gamma_{OH}$).However, because the bio-based polymers and the dye had similar functional groups, the FT-IR spectra partially overlapped and were very similar [44].

Similar functional groups were also found in chitosan and starch, so the FT-IR spectra of the chitosan–starch–dye, chitosan–starch, and dye (shown in Figure S9 of the Supplementary Materials) were once again very similar and overlapping. In the FT-IR spectrum of the control chitosan–starch film, the following characteristic absorption bands could be observed: 3281 cm^−1^ ($\nu_{OH}$), 2930 cm^−1^ ($\nu_{CH_{2}}^{as}$), 2877 cm^−1^ ($\nu_{CH_{2}}^{s}$), 1649 cm^−1^ ($\nu_{C=O}$ - amide I-chitosan), 1556 cm^−1^ ($\delta_{NH_{2}}$ and $\nu_{C-N}$ overlapping), 1149 cm^−1^ ($\nu_{COC}^{s}$), 999 cm^−1^ ($\nu_{COC}^{as}$). In the FT-IR spectrum of the chitosan–starch–dye film, three bands appeared, which were shifted 2931 cm^−1^ ($\nu_{CH_{2}}^{as}$), 2885 cm^−1^ ($\nu_{CH_{2}}^{s}$), 1647 cm^−1^ ($\nu_{C=O}$ - amide I-chitosan) [35,45].

The FT-IR spectra of the chitosan–PVA–dye and chitosan–PVA films showed absorption bands characteristic of the vibrational groups found in chitosan and PVA, which were attributed in the control chitosan–PVA film as follows: 3271 cm^−1^ ($\nu_{OH}$), 2937 cm^−1^ ($\nu_{CH_{2}}^{as}$), 2908 cm^−1^ ($\nu_{CH_{2}}^{s}$), 1651 cm^−1^ ($\nu_{C=O}$ - amide I-chitosan), 1558 cm^−1^ ($\delta_{NH_{2}}$ and $\nu_{C-N}$ overlapping), 1086 cm^−1^ ($\nu_{COC}^{as}$), 652 cm^−1^ ($\gamma_{OH}$). Due to the intra- and intermolecular interactions of the polymeric matrix with the dye, there were bands that were shifted in the FT-IR spectrum of the chitosan–PVA–dye film: 3257 cm^−1^ ($\nu_{OH}$), 1541 cm^−1^ ($\delta_{NH_{2}}$ and $\nu_{C-N}$ overlapping), 1084 cm^−1^ ($\nu_{COC}^{as}$), 669 cm^−1^ ($\gamma_{OH}$) (the FT-IR spectra of the chitosan–PVA–dye, chitosan–PVA, and dye are presented in Figure S10 of the Supplementary Materials) [41,46].

#### 3.4.2. UV-VIS

**Figure 5:**
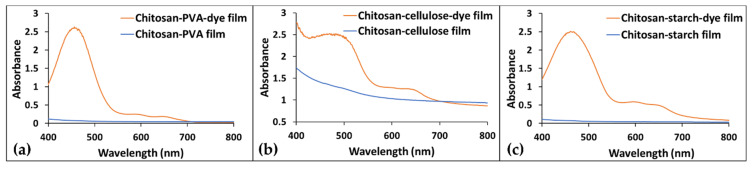
UV-VIS spectra of the bio-based polymeric films (control and with dye): (**a**) chitosan-PVA, (**b**) chitosan-cellulose and (**c**) chitosan-starch.

UV-VIS spectroscopy evidenced the incorporation of the dye in the polymeric matrices. The control films had no significant absorption in the visible region, as shown in Figure 5, whereas the bio-based polymeric films with added dye showed specific absorption maxima.

The chitosan–PVA–dye and the chitosan–starch–dye films (Figure 5a,c) had an absorption maximum at about 460 nm, while for the chitosan–cellulose–dye film (Figure 5b), the maximum was evidenced at 470 nm. In all of the UV-VIS spectra of the bio-based-dye-containing film, two shoulders could be observed around 575 and 650 nm.

#### 3.4.3. Thermal Analysis

The thermal properties of the bio-based polymeric films were investigated with thermogravimetric methods to determine the stability and decomposition temperature.

**Figure 6:**
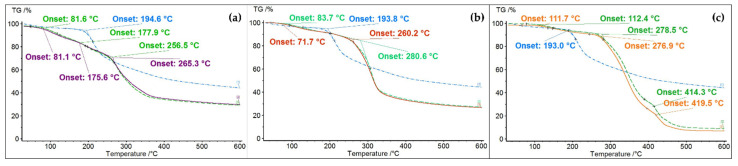
Thermograms of: (**a**) chitosan–cellulose–dye film (violet), chitosan–cellulose film (green), and dye (blue); (**b**) chitosan–starch–dye film (green), chitosan–starch film (red), and dye (blue); (**c**) chitosan–PVA–dye film (green), chitosan–PVA film (orange), and dye (blue).

In Figure 6, the thermograms of all obtained polymeric blends of the chitosan–cellulose–dye film, chitosan–cellulose film, and dye are presented. It can be observed that the polymeric films showed three stages of weight loss. In the temperature range of 25–200 °C, there were two weight losses attributed to the moisture loss from the surface water of the films and to the intrinsic water loss, with onsets at 81.6 °C (chitosan–cellulose film) and 177.9 °C (chitosan–cellulose–dye film). The film containing the dye exhibited similar thermal behavior, but the onsets were shifted to 81.1 and 175.6 °C. The most important mass loss occurred in the third stage, in the temperature range of 200–400 °C, with onsets at 256.5 °C for the chitosan–cellulose film and 265.3 °C for the chitosan–cellulose–dye film. Thermal degradation and decomposition occurred in this stage.

The mass losses of all obtained polymeric blends that occurred in different temperature ranges are presented in the Supplementary Materials, Tables S1–S3.

Comparing the thermograms of the chitosan–starch films with and without the dye with the thermogram of the bio-inspired dye, a better thermal stability of films up to 270 °C can be observed, while the degradation of the dye started after 200 °C. The thermograms of the polymeric films show two weight loss stages, which are indicated by the onset points at 83.7 and 280.6 °C for the chitosan–starch–dye film and at 71.7 and 260.2 °C for the chitosan–starch film. The first stage corresponded to the loss of surface moisture, while the second stage could be correlated to a degradation process of the films.

The chitosan–PVA films presented a thermal behavior similar to that of the chitosan–cellulose films due to the existence of three weight loss stages, but in this case, the third stage took place at higher temperature: 414.3 °C for the chitosan–PVA–dye film and 419.5 °C for the chitosan–PVA film. The first stage, which had its onset at 112.4 °C for the chitosan–PVA–dye film and at 111.7 °C for the chitosan–PVA film, corresponded to surface water vaporization, while the second stage, which had onsets at 278.5 °C for the chitosan–PVA–dye film and 276.9 °C for the chitosan–PVA film, was attributed to the degradation of chitosan and the dihydroxylation of PVA [41,47].

### 3.5. Films’ Sensitivity to pH Changes 

**Figure 7:**
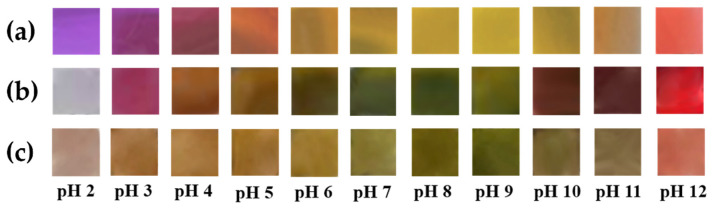
Bio-based-dye-containing polymeric films’ colors at different pH values: (**a**) chitosan–PVA–dye film, (**b**) chitosan–starch–dye film, and (**c**) chitosan–cellulose–dye film.

The sensitivity of the bio-based-dye-containing polymeric films to pH changes was evaluated through immersion in buffer solutions with pH values ranging from 2 to 12. Visible color changes were observed for all the tested samples after one hour of immersion, as shown in Figure 7.

The polymer matrix appeared to influence the color changes of the films due to the different intra- and intermolecular interactions with the dye.

The UV-VIS spectra after 1 h of immersion were recorded for both the polymeric films and the buffer solutions to evaluate desorption of the dye (Supplementary Materials, Figures S11–S13). For the chitosan–cellulose–dye, no desorption was shown, whereas for the chitosan–starch and chitosan–PVA dye-containing films, the UV-VIS spectra of the buffer solutions showed that the dye migrated from the polymeric matrix to the solution to some extent. In the pH ranges from 2 to 4 and from 10 to 12, greater desorption was observed than in the pH interval of 4 to 10. One possible cause could be the solubility of chitosan in acidic media, so the dye was forced out of the matrix. The desorption at high pH values could be explained by the dye’s greater solubility in alkaline media and/or because, at these pH values, the dye remained without protons, so it could not bind to the polymer matrix through H-bonds. In the 4 to 10 pH range, there was only a small desorption of the dye, which is a promising aspect because most of the pH changes caused by food spoilage occur in this interval. Based on the observed color changes, upon pH modification, the most suitable food products for this to be tested on could be milk, pork and chicken meat, and fish or shrimp. The pH indicators could be tested as freshness indicators for intelligent food packaging in different scenarios: they could be incorporated into conventional food packaging that has direct contact with the food (e.g., minced meat, chicken, and milk) and for the monitoring of the environment around the food (fish or shrimp).

For food packaging, the storage stability is one of the most important aspects to be studied. However, intelligent food packaging with freshness indicators can only be used for short-term storage due to the limited shelf life of the food products that these indicators monitor.

## 4. Conclusions

In this study, a new bio-inspired pH-sensitive dye was synthesized through acid-catalyzed condensation. The structural identity of the compound was elucidated with FT-IR and 1D and 2D NMR spectroscopic methods. The toxicity evaluation of the synthesized dye and its natural analogue, malvidin, which was carried out using theoretical methods, showed no toxicity risks, which is an essential requirement for food safety. Of course, experimental toxicity studies are mandatory and must be performed, but these are promising results that can lead to the creation of reproducible films that are satisfactory for industrial applications and that cannot be obtained with natural dye extracts or would be too expensive with pure anthocyanins. The bio-inspired dye exhibited pH-dependent photochromic properties, which were evidenced by the presence of multiple species at different pH values. A network of reversible chemical reactions for transformations upon pH changes was proposed. The dye was successfully incorporated into biodegradable intelligent systems based on chitosan blends, namely, chitosan–PVA, chitosan–cellulose, and chitosan–starch, respectively, which was proven by the absorption maxima corresponding to the dye in the UV-VIS spectra of the polymeric matrices. The thermal analysis of the pH indicator films showed that the chitosan–PVA–dye film exhibited the most thermal stability up to 300 °C. The sensitivity to pH variations was evaluated in buffer solutions over time, and visible color changes were observed. The perceived color variation of films in the pH range of 4 to 8 is essential for their application as freshness indicators. Moreover, in this pH domain, a minimal desorption of the dye was determined. Further studies will require testing of the films on food matrices; however, the key factor was for the films to exhibit pH-responsive behavior, since pH indicators based on these types of blends are widely tested on food with promising results as to their mechanical, thermal, and barrier properties, along with processability.

## Figures and Tables

**Table 1:** Calculated descriptors for malvidin and the synthesized dye structures.

	Mutagen	Tumoral	Irritating	Teratogen	cLogP	LogS	MW	TPSA	Drug-Likeness	Drug Score
Malvidin	-	-	-	-	1.84	−3.59	331	99.38	−3.35	0.43
Dye	-	-	-	-	3.59	−5.11	354	41.93	−1.89	0.35

## Supplementary Materials

The following supporting information can be downloaded at: https://www.mdpi.com/article/10.3390/polym14173622/s1, Figure S1: FT-IR spectrum of the synthesized dye; Figure S2: ^1^H-NMR spectrum of the bio-inspired dye; Figure S3: ^13^C-NMR spectrum of the bio-inspired dye; Figure S4: DEPT 135 spectrum of the synthetized dye; Figure S5: HMBC spectrum of the synthesized dye; Figure S6: UV-VIS spectrum of the dye; Figure S7: Thermogram of the dye; Figure S8: UV-VIS spectra of dye solutions (7∙10^–5^ M methanol: water 1:14) at pH values ranging from 2 to 12 over time; Figure S9: FT-IR spectra of chitosan–starch–dye (green), chitosan–starch (blue), and dye (red); Figure S10: FT-IR spectra of chitosan–PVA–dye (green), chitosan–PVA (blue), and dye (red); Figure S11: UV-VIS spectra of the chitosan-starch-dye films after 1 h in buffer solutions a) and of the buffer solutions b); Figure S12: UV-VIS spectra of the chitosan–PVA–dye film after 1 h in buffer solutions a) and those of the buffer solutions b); Figure S13: UV-VIS spectra of the chitosan–cellulose–dye film after 1 h in buffer solutions; Table S1: Weight losses of the chitosan–cellulose–dye film, chitosan–cellulose film, and the dye; Table S2: Weight losses of the chitosan–starch–dye film, chitosan–starch film, and the dye; Table S3: Weight losses of the chitosan–PVA–dye film, chitosan–PVA film, and the dye.
