# Supplementary material for: Bio-Based pH Indicator Films for Intelligent Food Packaging Applications
Source: Polymers (Basel). 2022 Sep 1;14(17):3622. doi: 10.3390/polym14173622 (PMC9460188; doi:10.3390/polym14173622)
Supplement: Supplementary file 1 [file polymers-14-03622-s001.zip › polymers-1878108-supplementary.pdf]

Supplementary Materials
Bio-based pH indicator films for intelligent food packaging applications

Iulia Păușescu[1], Diana-Maria Dreavă[1], Ioan Bîtcan[1], Raluca Argetoianu[1], Diana Dăescu[1], Mihai Medeleanu[1, *]

[1]University Politehnica Timişoara, Faculty of Industrial Chemistry and Environmental Engineering, Carol Telbisz 6, 300001 Timisoara, Romania
* *mihai.medeleanu@upt.ro*

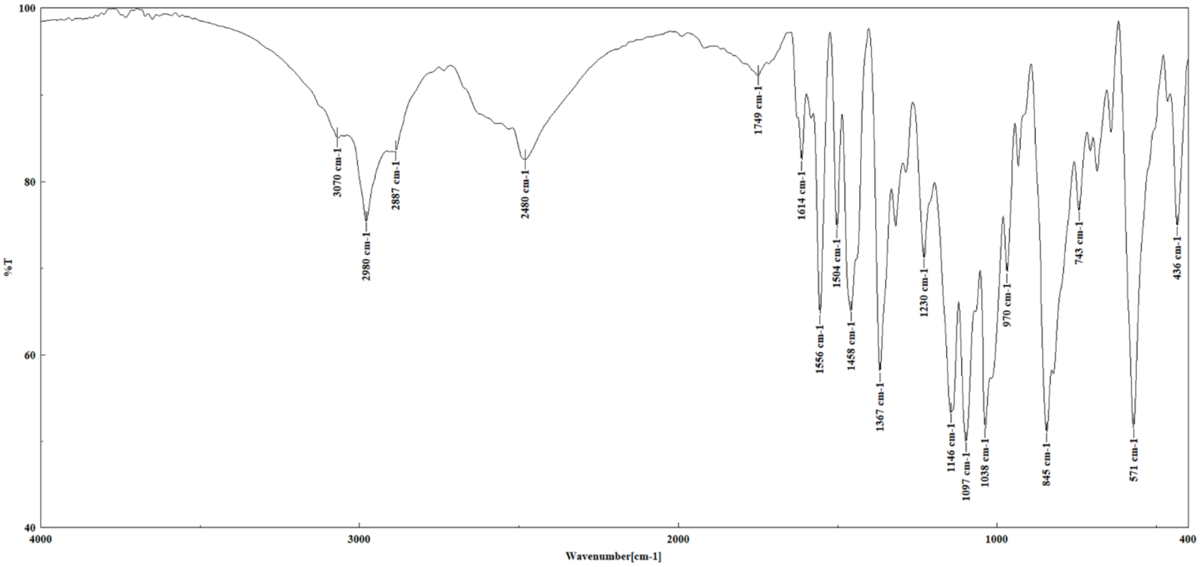

Figure S1. FT-IR spectrum of the synthesized dye

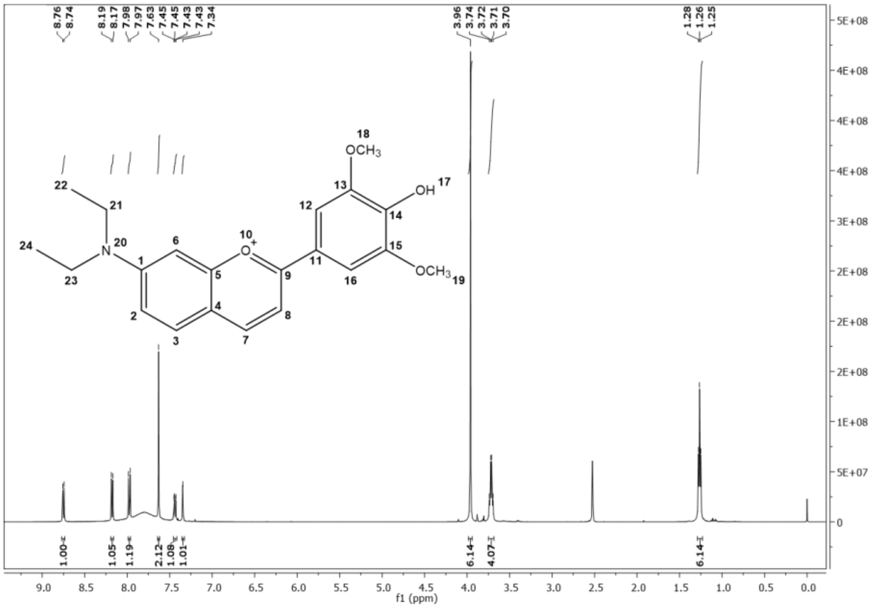

Figure S2. ^{1}H-NMR spectrum of the bio-inspired dye

^{1}H-RMN (500 MHz, DMSO-d6, δ ppm): 1.26 (t, 6H, *J*=7.0 Hz, H22, H24); 3.72 (q, 4H, *J*=7.0 Hz, H21, H23); 3.96 (s, 6H, H18, H19); 7.34 (s, 1H, H6); 7.44 (dd, 1H, *J*=9.4; 2.3 Hz, H2); 7.63 (s, 2H, H12, H16); 7.98 (d, 1H, *J*=9.4 Hz, H3); 8.18 (d, 1H, *J*=8.3 Hz, H8); 8.75 (d, 1H, *J*=8.3, H7).

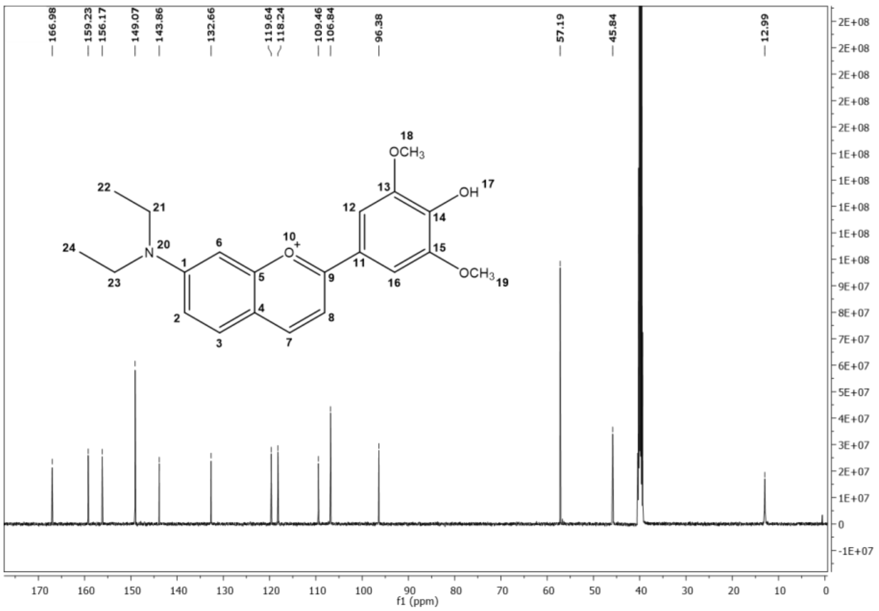

Figure S3. ^{13}C-NMR spectrum of the bio-inspired dye

^{13}C-RMN (125 MHz, DMSO-d6, δ ppm): 12.9 (C22, C24); 45.8 (C21, C23), 57.1 (C18, C19); 96.3 (C6); 106.8 (C12, C16); 109.4 (C8), 118.2 (C2), 119.6 (C4, C11); 132.6 (C3), 143.8 (C14); 149.0 (C7, C13, C15); 156.1 (C1); 159.2 (C5); 166.9 (C9).

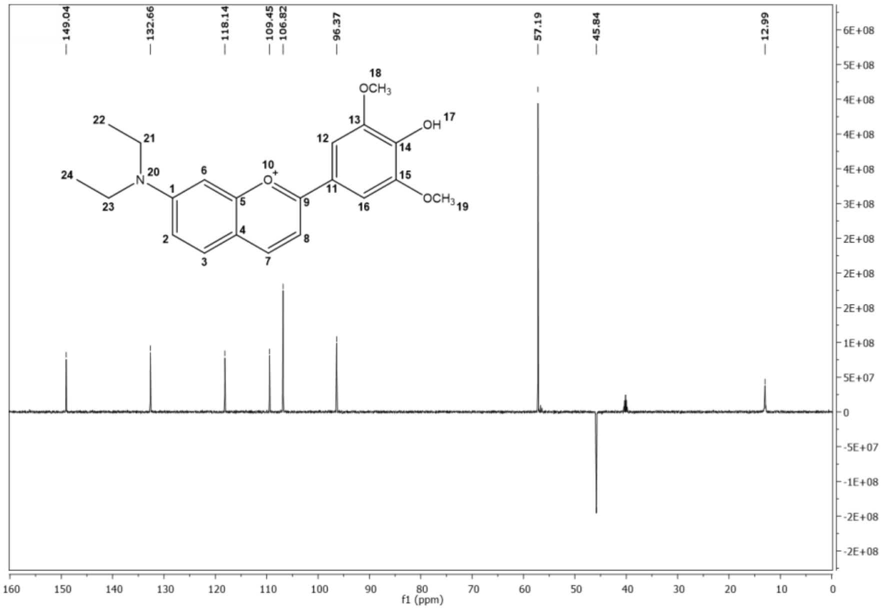

Figure S4. DEPT 135 spectrum of the synthetized dye

45.8 (C21, C23 – secondary carbon atoms), 57.1 (C18, C19 – tertiary carbon atoms), 96.3 (C6 - tertiary carbon atom); 106.8 (C12, C16 – tertiary carbon atoms); 109.4 (C8 – tertiary carbon atom), 118.2 (C2– tertiary carbon atom), 132.6 (C3 tertiary carbon atom), 149.0 (C7 tertiary carbon atom).

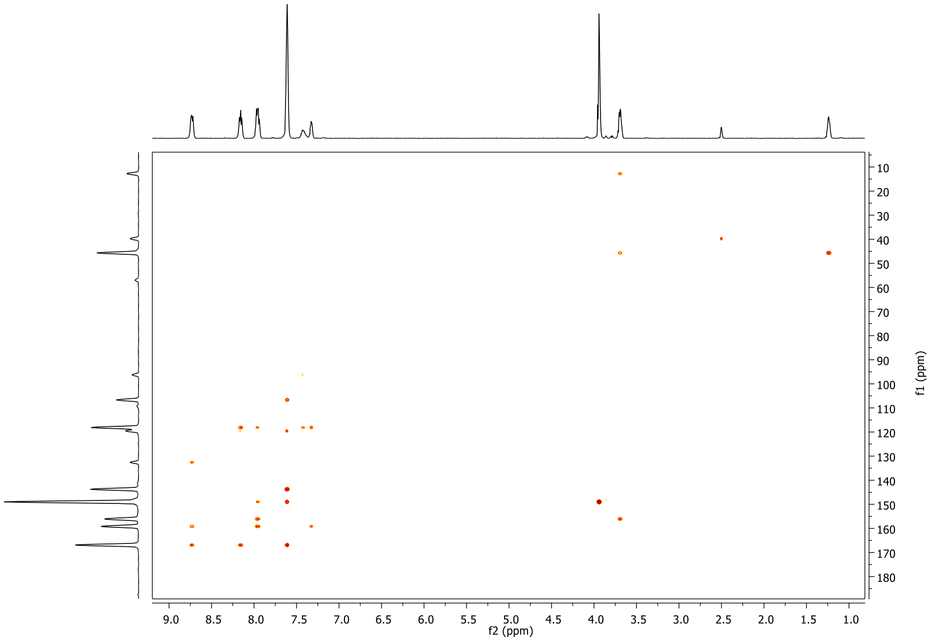

Figure S5. HMBC spectrum of the synthetized dye

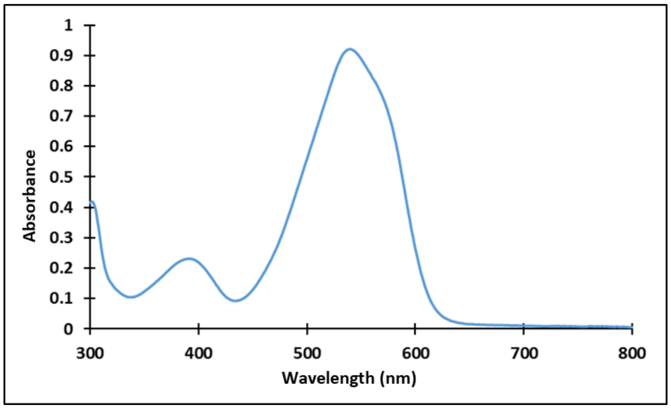

Figure S6. UV-VIS spectrum of the dye

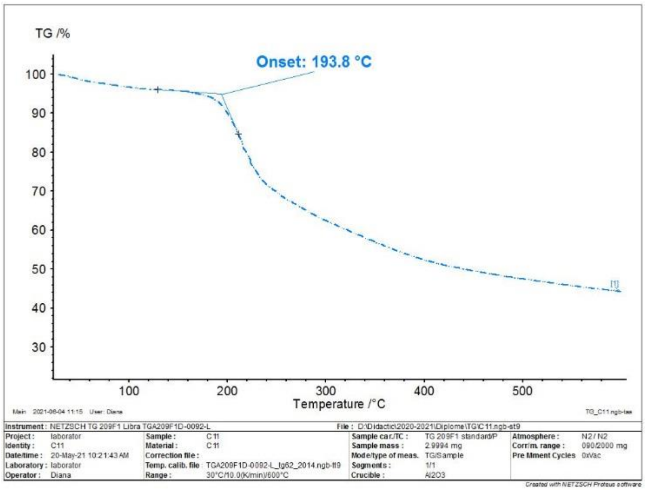

Figure S7. Thermogram of the dye

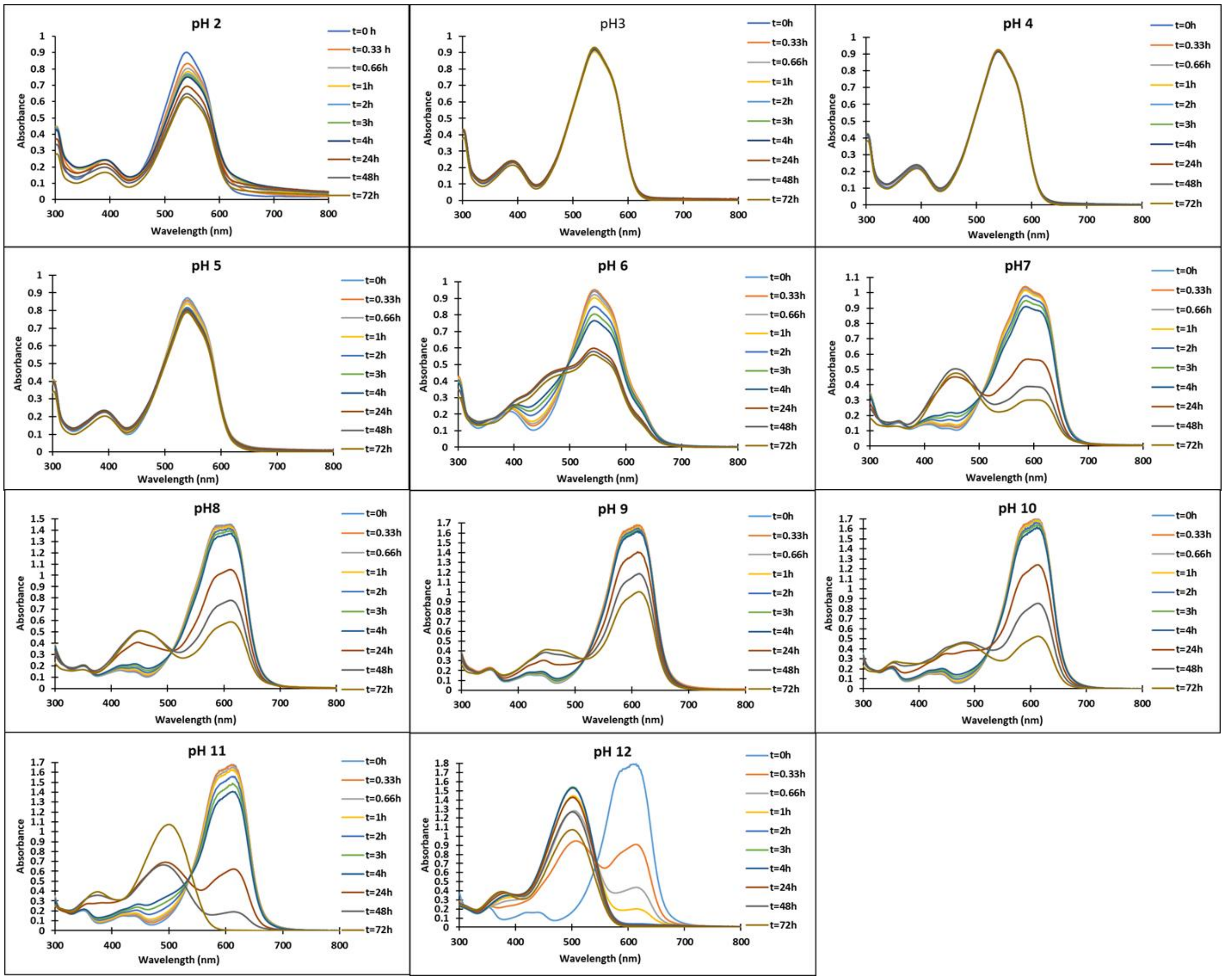

Figure S8. UV-VIS spectra of dye solutions ($7{\cdot}10^{-5}$ M methanol: water 1:14) at pH ranging from 2 to 12 over time

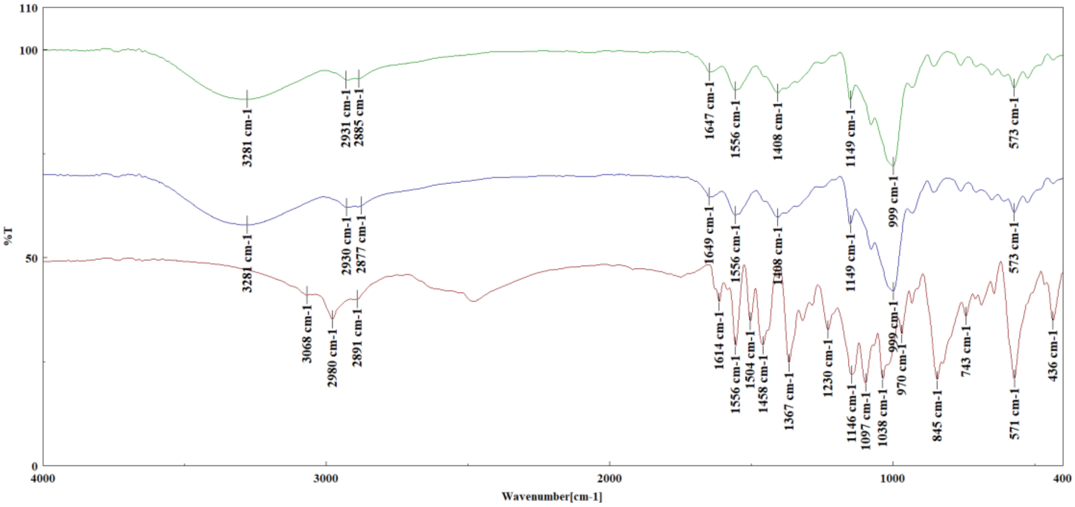

Figure S9. FT-IR spectra of chitosan-starch-dye (green), chitosan-starch (blue) and dye (red)

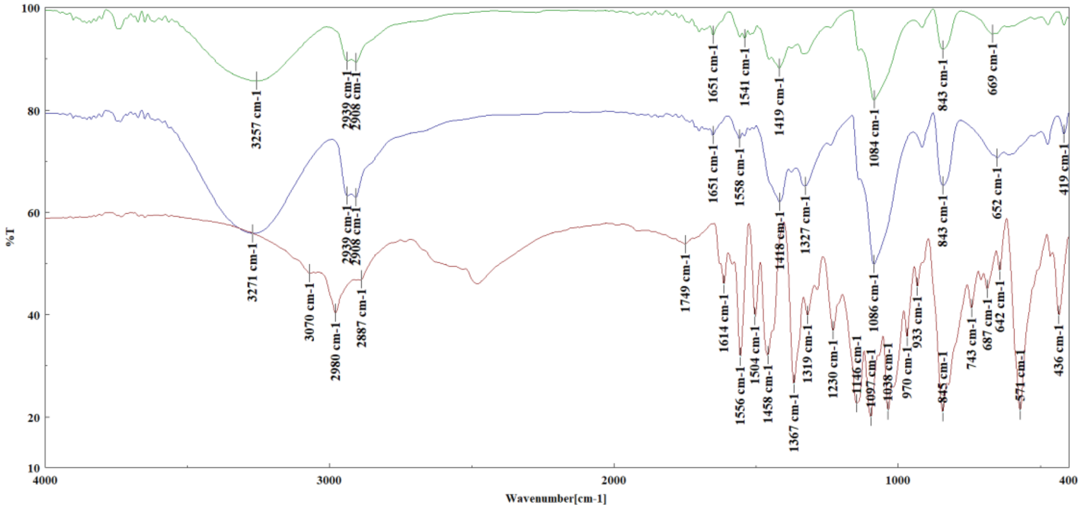

Figure S10. FT-IR spectra of chitosan-PVA-dye (green), chitosan-PVA (blue) and dye (red)

Table S1. Weight losses of the chitosan-cellulose-dye film, chitosan-cellulose film and of the dye

Compound	Weight loss [%]				
	25-200°C	25-300°C	25-400°C	25-500°C	25-600°C
chit-cell-dye	18.71	46.06	63.37	66.59	68.64
chit-cell	20.65	49.09	65.89	68.90	70.84
dye	9.91	37.52	47.62	52.52	55.78

Table S2. Weight losses of the chitosan-starch-dye film, chitosan-starch film and of the dye

Compound	Weight loss [%]				
	25-200°C	25-300°C	25-400°C	25-500°C	25-600°C
chitosan-starch-dye	9.32	34.24	66.16	70.63	72.81
chitosan-starch	9.15	37.32	67.02	71.29	73.30
dye	9.91	37.44	47.54	52.44	55.69

Table S3. Weight losses of the chitosan-PVA-dye film, chitosan-PVA film and of the dye

Compound	Weight loss [%]				
	25-200°C	25-300°C	25-400°C	25-500°C	25-600°C
chitosan-PVA-dye	6.69	19.41	68.07	89.99	91.14
chitosan-PVA	7.63	11.12	74.45	92.39	93.14
dye	9.91	37.52	47.62	52.52	55.78

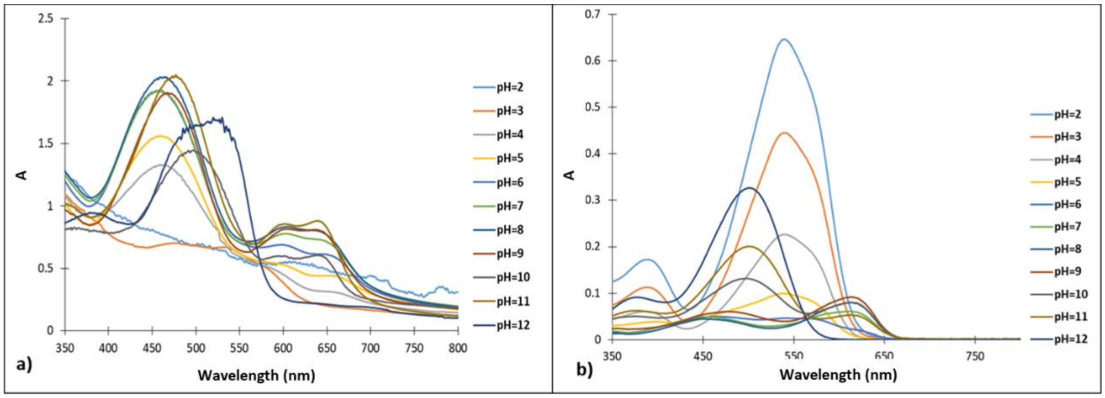

Figure S11. UV-VIS spectra of the chitosan-starch-dye films after 1h in buffer solutions a) and of the buffer solutions b)

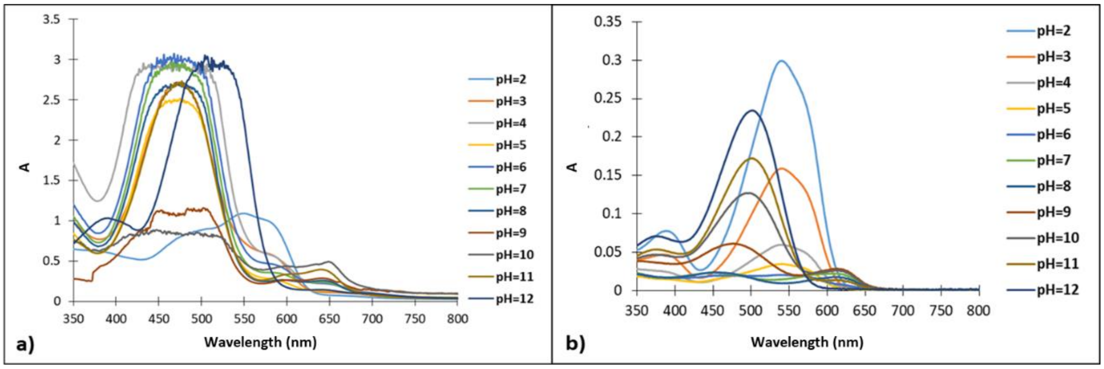

Figure S12. UV-VIS spectra of the chitosan-PVA-dye film after 1h in buffer solutions a) and of the buffer solutions b)

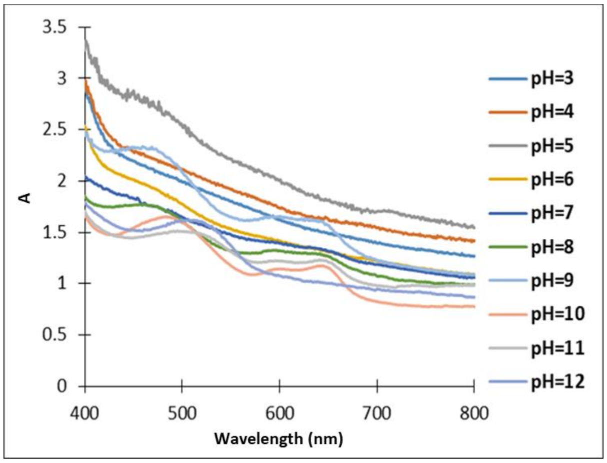

Figure S13. UV-VIS spectra of the chitosan-cellulose-dye film after 1h in buffer solutions
